# Natural and anthropogenic landscape factors shape functional connectivity of an ecological specialist in urban Southern California

**DOI:** 10.1111/mec.16656

**Published:** 2022-09-13

**Authors:** Sarah M. Wenner, Melanie A. Murphy, Kathleen S. Delaney, Gregory B. Pauly, Jonathan Q. Richmond, Robert N. Fisher, Jeanne M. Robertson

**Affiliations:** ^1^ Department of Biology California State University Northridge California USA; ^2^ Department of Ecosystem Science and Management, Program in Ecology University of Wyoming Laramie Wyoming USA; ^3^ National Park Service Thousand Oaks California USA; ^4^ Natural History Museum of Los Angeles County Los Angeles California USA; ^5^ US Geological Survey Western Ecological Research Center San Diego California USA

**Keywords:** conservation genetics, habitat degradation, landscape genetics, Phrynosoma, population genetics, reptiles, wildlife management

## Abstract

Identifying how natural (i.e., unaltered by human activity) and anthropogenic landscape variables influence contemporary functional connectivity in terrestrial organisms can elucidate the genetic consequences of environmental change. We examine population genetic structure and functional connectivity among populations of a declining species, the Blainville's horned lizard (*Phrynosoma blainvillii*), in the urbanized landscape of the Greater Los Angeles Area in Southern California, USA. Using single nucleotide polymorphism data, we assessed genetic structure among populations occurring at the interface of two abutting evolutionary lineages, and at a fine scale among habitat fragments within the heavily urbanized area. Based on the ecology of *P. blainvillii*, we predicted which environmental variables influence population structure and gene flow and used gravity models to distinguish among hypotheses to best explain population connectivity. Our results show evidence of admixture between two evolutionary lineages and strong population genetic structure across small habitat fragments. We also show that topography, microclimate, and soil and vegetation types are important predictors of functional connectivity, and that anthropogenic disturbance, including recent fire history and urban development, are key factors impacting contemporary population dynamics. Examining how natural and anthropogenic sources of landscape variation affect contemporary population genetics is critical to understanding how to best manage sensitive species in a rapidly changing landscape.

## 1 INTRODUCTION

A major question in conservation management is how evolutionary, ecological, and anthropogenic processes shape contemporary patterns of functional connectivity (Allendorf et al., 2013). Functional connectivity, the degree to which organisms can effectively disperse through the landscape, reduces the impact of stochastic processes (e.g., genetic drift) and promotes the long‐term persistence of populations (Crooks & Sanjayan, 2006; Frankham et al., 2010; Taylor et al., 1993; Tischendorf & Fahrig, 2000; Wiens & Milne, 1989). Contemporary functional connectivity reflects an interplay between barriers produced from natural (i.e., unaltered by human activity) landscape features, some of which have developed over geological times scales, and landscape features created by anthropogenic disturbance. Species respond to these environmental features differently based on their natural history (Miles et al., 2019). Thus, identifying how natural environmental factors and anthropogenic disturbance influence the functional connectivity of populations is critical for developing management strategies for species occupying urbanizing regions (Balkenhol et al., 2016; Funk et al., 2005; Lindenmayer et al., 2007; Tassone et al., 2020; Zellmer & Knowles, 2009).

Genomic signals often reflect features of the landscape and/or climate that have been in place long before human disturbance, such as topography and climatic gradients. At broad spatial and temporal scales, phylogeographical patterns often correlate to historical and ecological factors such as historical geography and dispersal events (Avise, 2000; Rissler, 2016; Sanmartín, 2012). At fine spatial scales, the ecology of a species determines how environmental heterogeneity affects presence, movement, and gene flow (Engler et al., 2014; Keyghobadi et al., 2005; Robertson et al., 2018; Sánchez‐Montes et al., 2017; Trumbo et al., 2021; Zero et al., 2017). Specifically, species with short dispersal distances, limited mobility, and strong microhabitat preferences are expected to be more limited by natural environmental barriers and may be particularly sensitive to the addition of anthropogenic barriers.

Anthropogenic disturbances have direct and indirect effects on connectivity through habitat fragmentation and degradation (e.g., vegetation community conversion and introduction of invasive species). Urban development and land use change can act as a barrier or a conduit to gene flow, depending on the species and the composition of the urban landscape (Ballare & Jha, 2020; Miles et al., 2019). Species with limited dispersal abilities, as well as those incapable of occupying the urban matrix, exhibit higher levels of genetic divergence in response to anthropogenic factors including habitat fragmentation and habitat loss (Delaney et al., 2010; Epps & Keyghobadi, 2015; Miles et al., 2019; Richmond et al., 2009). Disturbances can rapidly reduce movement and gene flow across the landscape, resulting in smaller population sizes, lower levels of genetic diversity, and amplified patterns of genetic spatial structure over short time periods (Epps & Keyghobadi, 2015; Fahrig, 2003; Keyghobadi, 2007; Ovaskainen & Hanski, 2004; Tassone et al., 2020). In some cases, population structure does not reflect contemporary barriers due to a temporal lag between barrier formation and resulting changes in genetic signature (Bolliger et al., 2014; Epps & Keyghobadi, 2015; Landguth et al., 2010; Richmond et al., 2017; Spear & Storfer, 2008). Even so, examining the effects of contemporary disturbance regimes on genetic structure and connectivity of populations is a key step for the conservation of biodiversity in urbanizing regions.

The Southern California/northern Baja California Coast Ecoregion is a well‐known hotspot of biodiversity, with numerous endemic and imperilled species threatened by urban‐related disturbance (Brooks et al., 2006; Mittermeier, 2004; Myers et al., 2000). One example is the Blainville's horned lizard (*Phrynosoma blainvillii*), a Priority II Species of Special Concern in California due to range wide decline (Jennings & Hayes, 1994; Thomson et al., 2016). *Phrynosoma blainvillii* is a dietary specialist with a small dispersal distance and home‐range size that varies considerably by year and season (0.0058–0.14 km^2^) (Alberts et al., 2004; Fisher et al., 2002; Hult & Germano, 2015). Additionally, this species prefers loose, sandy soils for burrowing, burrows and shrubs for refugia, and open areas for thermoregulation (Alberts et al., 2004; Fisher et al., 2002; Hult & Germano, 2015). The primary prey of *P. blainvillii* is native harvester ants (e.g., *Pogonomyrmex*, *Messor* spp., and others), which play an important role in seed dispersal and redistribution of soil and nutrients (Alberts et al., 2004; Fisher et al., 2002; Gosselin et al., 2016; Pianka & Parker, 1975). Unfortunately, Southern California has been subject to invasion by the Argentine ant (*Linepithema humile*), which displaces native ant species, alters faunal and floral community structure, and reduces habitat quality (Richmond et al., 2021; Suarez et al., 1998, 2000; Suarez & Case, 2002). *Phrynosoma blainvillii* is unable to substitute the native ant prey base with the Argentine ant and exhibits significantly decreased growth rates and survival in the presence of this invader (Fisher et al., 2002; Suarez & Case, 2002).

There are three lineages of *Phrynosoma blainvillii*: Northern California, Southern California, and Northern Baja California (Leaché et al., 2009). The boundary between the Northern California and Southern California lineages falls within northwestern Los Angeles County, CA. In Southern California, this species has experienced an estimated 45% reduction in its distribution, primarily due to anthropogenic disturbances including habitat loss due to landscape change, pesticide use, the Argentine ant invasion, and over‐collecting (Jennings, 1987; Jennings & Hayes, 1994; Thomson et al., 2016). A reduction in coastal sage scrub and chaparral has left many populations isolated within habitat patches; therefore, understanding patterns of functional connectivity among the remaining populations is important for future management of this species.

Our goal was to inform management of a species of conservation concern by examining population genetic structure and drivers of functional connectivity across the study region. First, we investigated population structure at two spatial scales, and focused on the boundary of the Northern and Southern California lineages of *P. blainvillii* (Leaché et al., 2009). This permitted us to pinpoint the geography of the contact zone and measure the extent of admixture between the two lineages. Across the entire study region, multiple *P. blainvillii* populations are isolated by extensive urbanization. Therefore, we predicted relatively low levels of connectivity among sampling sites due to the life history of the animals (see above), and high levels of genetic divergence among isolated habitat fragments due to contemporary barriers to connectivity. However, we expected to find instances of shared ancestral polymorphism despite lack of current connectivity due to the recency of these barriers. Second, we generated hypotheses for the ecological factors that limit or promote functional connectivity among populations of *P. blainvillii* (Table 1). We predicted that due to the general thermal requirements of ectotherms and the strong ecological preferences of *P. blainvillii*, some environmental factors, including vegetation community, microclimate, soil composition, and topography, have influenced functional connectivity over geologic time scales. We examined these natural landscape factors along with factors produced by anthropogenic disturbance, including recent fire history and urban development, to inform management strategies in an urban‐compromised landscape.

**TABLE 1:** Landscape variables used for testing gravity models, predicted effects, and brief ecological justifications for the effect on functional connectivity of *Phrynosoma blainvillii*. Landscape processes are independent hypotheses and are tested in individual models. Parameters are estimated in the gravity equation: Geographic distance (w), production (v), and resistance (c). Data sources are open source, and the corresponding resolution of each variable is included.

Parameter	Process (hypothesis)	Variable	Code	Data source	Resolution	Prediction	Ecological justification
*v*	Fine‐scale topography (at)	Surface relief ratio (3 × 3 cell window)	srr3	DEM (SRTM)	30 m	−	Horned lizards are small‐bodied and limited by complex topographies
*v*	Microclimate (at)	Annual dryness index	adi	WorldClim	1 km	+	Horned lizards prefer a drier microclimate
*v*	Soil type (at)	Percent sand in topsoil (0–5 cm)	sand	ISRIC	250 m	+	Horned lizards prefer loose, sandy soil (Fisher et al., 2002; Pianka & Parker, 1975)
*v*, *c*	Vegetation (at, btw)	Percent of land cover with shrub & scrub (shrub height <5 m)	shrub	NLCD 2016	30 m	+	Strong habitat preference for native chaparral shrub communities (Alberts et al., 2004; Fisher et al., 2002; Hult & Germano, 2015)
		Compound topographic index	cti	DEM (SRTM)	30 m	+	Wetness increases productivity of the vegetation in a site
*v*, *c*	Thermoregulation (at, btw)	Heat load index	hli	DEM (SRTM)	30 m	+	Ectotherms require warmth to perform
*c*	Anthropogenic development (btw)	Impervious surfaces	imperv	NLCD 2016	30 m	−	Areas with a high proportion of impervious surfaces are not used by horned lizards
*c*	Fire history (btw)	Time since last fire	fire	NPS		−	Fire opens up habitat facilitating movement and gene flow of horned lizards (Roche 2010)
*c*	Broad‐scale topography (btw)	Surface relief ratio (27 × 27 cell window)	srr27	DEM (SRTM)	30 m	−	Horned lizards have small ranges and are unable to navigate complex topographies
*w*	Null (distance)	Topographic distance (Euclidean)	length			−	Isolation by distance

## 2 MATERIALS AND METHODS

### 2.1 Study area and field collection

**FIGURE 1:**
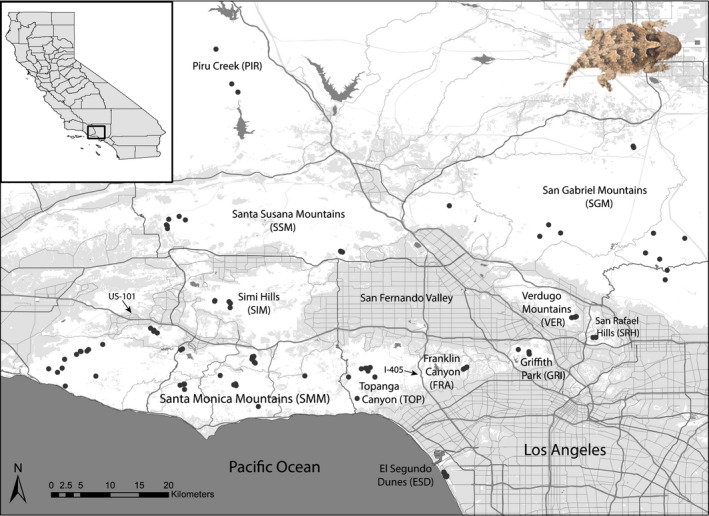
*Phrynosoma blainvillii* sampling sites and delineation of geographic regions within Los Angeles and Ventura counties in Southern California. Shading corresponds to land cover data: Undeveloped parks/natural open space (white); and commercial, industrial, residential, agricultural, and developed parks/other open areas (gray). Major roadways indicate habitat fragmentation of the region. Data sources: Southern California Association of Governments (2016) and Geographic Data Technology, Inc.

We implemented an individual‐based sampling scheme to examine *P. blainvillii* from areas spanning the contact zone between the Northern and Southern California lineages in Southern California (Figure 1, Table S1). Many of the samples were collected as part of long‐term monitoring by Santa Monica Mountains National Recreation Area, resulting in intensive sampling in the Santa Monica Mountains (SMM) and the Simi Hills (SIM). We collected additional samples from the Santa Susana Mountains (SSM), San Gabriel Mountains (SGM), Verdugo Mountains (VER), San Rafael Hills (SRH), Piru Creek (PIR), and El Segundo Dunes (ESD). There is extensive urban development within and among the mountain ranges (Figure 1). Our sampling from the western Santa Monica Mountains and Piru Creek overlap with the southernmost samples in the Northern California lineage described by Leaché et al. (2009); their samples VNTR1–4 and LA 2, respectively. Our easternmost samples from the San Gabriel Mountains are approximately 35 km west of the northernmost sample in the Southern California lineage described by Leaché et al. (2009; their sample LA 1), which was from the desert slope of this mountain range. We hand‐captured lizards, used nonlethal sampling methods to collect tissue samples (tail or toe clip), and recorded latitude and longitude coordinates in decimal degrees to the nearest hundred‐thousandth using a handheld GPS device.

### 2.2 Genomic DNA extraction and library preparation

We extracted genomic DNA using a Qiagen DNeasy blood and tissue extraction kit (Qiagen). We used RAD‐sequencing to develop a SNP data set following the BestRAD library preparation protocol (Ali et al., 2016). We began by digesting 50 ng of genomic DNA from each sample with the restriction enzyme Sbf1. Then, we ligated unique BestRAD barcodes onto each sample, pooled the samples into a single library, used sonication to shear to 400–600 bp lengths, and ligated Illumina sequencing adaptors. Lastly, we performed a PCR and used Ampure bead cleanups to size‐select for 500 bp length fragments. Libraries were sent to Admera Health through Genohub Inc. for sequencing on an Illumina HiSeq 4000. Sequences were 150 bp long and paired‐end with 72 samples per lane.

### 2.3 Demultiplexing and de novo assembly

We demultiplexed each lane using the process_radtags program in stacks version 2.0 (Catchen et al., 2011, 2013; Rochette et al., 2019). We calculated the percent‐retained reads per individual as an initial assessment of alignment and conducted de novo assembly using the wrapper denovo_map.pl (Rochette & Catchen, 2017). We randomly selected 40 individuals from across the sampling region to build the catalogue of loci using cstacks under a range of parameters: *M & n* from 1 to 9 (Catchen et al., 2011, 2013; Rochette et al., 2019), and then selected parameter values based on the number of polymorphic loci shared by 80% of samples (r80 loci) and the distribution of the number of SNPs per locus (Rochette & Catchen, 2017). The final parameter values were a minimum stack depth of 3 (*m* parameter), maximum three mismatches per locus within an individual (*M* parameter), and three mismatches between loci across different individuals in the global data set (*n* parameter). Finally, we mapped scaffolds for all individuals against the catalogue using sstack*s* (Catchen et al., 2011, 2013; Rochette et al., 2019).

### 2.4 Population filtering

We applied population filters using VCFtools (Danecek et al., 2011) and implemented an iterative filtering strategy to maximize the number of retained individuals and SNPs (O'Leary et al., 2018). Individuals with >95% missing data were removed before subsequent filtering. Next, we removed loci with 70% or more missing data and a minor allele frequency of 0.05, and filtered genotypes by a maximum depth of 35. Indels were filtered out within the stacks pipeline.

### 2.5 Assessing population structure and genetic variation

We first identified geographic areas for population‐based analyses by mountain range and then by extent of urban isolation, such that “geographic area” refers to the following: Santa Monica Mountains (SMM), Simi Hills (SIM), Franklin Canyon (FRA), Griffith Park (GRI), Santa Susana Mountains (SSM), Piru Creek (PIR), San Gabriel Mountains (SGM), Verdugo Mountains (VER) and El Segundo Dunes (ESD). Topanga Canyon (TOP) was combined with the SMM samples due to proximity. Additionally, we combined the Verdugo Mountains and San Rafael Hills samples due to their close geographic proximity and the small sample size from the San Rafael Hills (Figure 1).

To examine genetic relationships within and among these geographic areas, we used a principal components analysis of Euclidean distances between individual genotypes in plink version 1.90. Principal components were color‐coded by geographic area and visualized with 95% confidence intervals in ggplot2 in R version 3.6.2 (R Core Team, 2013; Purcell et al., 2007; Wickham, 2016). PCA is unaffected by uneven sample sizes per geographic area and provides a good first look at the genetic variation among individuals (Benestan et al., 2016).

We used structure to determine the number of demes and explore patterns of admixture at multiple scales (Falush et al., 2003; Pritchard et al., 2000). For each structure run, we implemented 20 iterations of *K* = 1–10 with 10,000 burnin and 10,000 MCMC replicates and checked alpha values across runs to confirm convergence (Benestan et al., 2016; Evanno et al., 2005; Gilbert et al., 2012). We used the Evanno method in structure harvester (Earl & vonHoldt, 2012; Evanno et al., 2005) to determine likelihood scores for each *K* value, and the Large *K* Greedy algorithm in clumpak (Kopelman et al., 2015) to align the assignment matrices for all structure runs at each *K* value. We created assignment plots using clumpak (Kopelman et al., 2015) and plotted the estimated assignment coefficients in ArcMap 10.6 for better visualization of spatial relationships. As an additional measure of genetic connectivity, we estimated genetic relatedness among geographic regions by calculating pairwise population differentiation with 9999 permutations of the *F*_ST_ statistic in genodive (Meirmans & Van Tienderen, 2004). Lastly, we performed an AMOVA in genodive with 9999 permutations to determine the distribution of genetic variation within and among the geographic regions (Meirmans & Van Tienderen, 2004).

### 2.6 Landscape genomics

To examine the effect of the environment on functional connectivity, we selected landscape variables suitable for assessing our hypotheses based on the species' natural history and ecology (Table 1). Our hypotheses considered both natural and anthropogenic sources of environmental variation, including elements of topography at fine and broad scales, microclimate, time since fire, urban development, vegetation community, and sand content in the soils in two contexts: “at‐site” and “between‐site” (Table 1). At‐site variables indicate the characteristics of a horned lizard's preferred microhabitat that correlate to the production of offspring, which then move and/or disperse through the landscape. The production of higher numbers of offspring, and therefore potential migrants, increases the functional connectivity among sites. Between‐site variables represent the environmental characteristics that promote or impede movement across the landscape. Lastly, we expected to find a positive relationship between geographic and genetic distance due to isolation by distance.

We hypothesized that the important at‐site conditions for horned lizard productivity are low complexity fine‐scale topography, high sand content of the topsoil, native shrub vegetation, and a warm and dry microclimate. As a measure of fine‐scale topographic complexity, we used surface relief ratio (Pike & Wilson, 1971) at 3‐m (srr3) resolution. As a measure of microclimate, we selected the annual dryness index (Rehfeldt, 2006), which is a proxy for the amount an area dries out due to heat. Annual dryness index is calculated by dividing the degree‐days >5 (dd5—based on mean monthly temperature) by mean annual precipitation (Baskerville & Emin, 1969; Rehfeldt, 2006; Rehfeldt et al., 2009; Zalom et al., 1983). We used mean percent sand in the topsoil (0–5 cm) as an assessment of soil type preference. To test the importance of chaparral habitat, we used two parameters, percent land cover containing woody shrub/scrub (shrubs <5 m tall) and compound topographic index (cti), a measure of moisture. We used a measurement of incident radiation, the heat load index (hli), as a proxy for sun exposure necessary for thermoregulation.

We hypothesized that native chaparral vegetation for cover and heat for thermoregulation were also important between‐site characteristics facilitating migration, in addition to shorter amounts of time since fire (resulting in less dense vegetation and more open habitat), less complex broad‐scale topography, and minimal urban development. The same variables used to represent native chaparral vegetation and heat for thermoregulation for the at‐site conditions were calculated for the corresponding between‐site conditions. We estimated the time since the last fire by subtracting the year of the most recent burn from the current year. We used surface relief ratio (Pike & Wilson, 1971) at 27‐m (srr27) resolution for broad‐scale topography. Lastly, we used a measure of imperviousness of the ground surface, which is the percentage of developed surface over each 30‐m pixel, to capture urban development of any given area.

The landscape data came from the 2016 National Land Cover Database (Homer et al., 2020), Shuttle Radar Topography Mission (SRTM, Farr et al., 2007) digital elevation model (USGS EROS Data Center), WorldClim climate data (Fick & Hijmans, 2017), SoilGrids 250‐m accessed through World Soil Information‐ISRIC (Hengl et al., 2017), and fire perimeters from the National Park Service (Table 1). We resampled all rasters to achieve 30‐m resolution. We calculated surface relief ratio (Pike & Wilson, 1971) at 3‐ and 27‐m scales, compound topographic index (Moore et al., 1991), and heat load index (McCune & Keon, 2002) from the digital elevation model (USGS EROS Data Center) using the spatialEco version 1.3–2 package in R version 3.6.2 (Evans, 2020; R Core Team, 2013).

We applied gravity models in the R package GeNetIt version 0.1–2 (Evans & Murphy, 2020; R Core Team, 2013). Gravity models are network models, which include two main components to form a network: points (individual lizards sampled) and edges (straight lines between points used to sample the landscape conditions between observations). Edges are used as a sample of the landscape and do not represent a movement path for individual animals (Murphy, Evans, & Storfer, 2010). The gravity model equation includes three terms: “v” the landscape variables that promote production (i.e., reproduction); “c” the landscape variables that increase resistance to gene flow, and “w” a spatial autocorrelation term. We natural‐log transformed the data and used singly‐constrained gravity models to account for nonindependence of pairwise comparisons (Fotheringham & O'Kelly, 1989; Murphy, Dezzani, et al., 2010). We calculated Euclidean distance between genotypes using the R package ECODIST (Goslee & Urban, 2007; R Core Team, 2013) and used 1 – Euclidean distance as a measure of gene flow (Shirk et al., 2017). We included geographic distance in every model because spatial autocorrelation is a requirement of the gravity model equation (Murphy, Dezzani, et al., 2010). We solved the gravity models as mixed effects linear models using maximum likelihood (Zuur et al., 2009). We treated landscape variables and geographic distance as fixed effects and sites as random effects (Murphy, Evans, & Storfer, 2010). To ensure that edges in the model sufficiently capture the true environmental variation in the landscape, we buffered the nodes and edges (30‐m native cell size; 90‐, 250‐, and 500‐m buffer sizes) in GeNetIt and then examined pairwise correlations between buffer widths (Evans & Murphy, 2020; Watts et al., 2015). We calculated statistical moments (mean, median, minimum, maximum, variance, kurtosis, skewness, and 75, 90, and 95% quantiles) at nodes and along edges at all buffer sizes for each of the landscape variables except shrub cover, which was calculated as a percent of cells between sites classified as shrub. Node and edge values were highly correlated across all buffer widths (*r* > 0.80), so native resolution (30‐m) values for both at‐ and between‐site variables were used in the models (as in Parsley et al., 2020; Robertson et al., 2018; Watts et al., 2015). Therefore, at‐site variables consisted of a point value, and between‐site variables were represented by the median values of the edges. We selected the median value due to its resistance to skewed distributions and outliers. Lastly, we tested for correlated variables using a threshold of *r* < 0.70 in the R package corrplot (Figure S1; R Core Team, 2013; Wei, 2017).

We tested pruned networks at 50‐ and 10‐km maximum distance between observations. We implemented a hierarchical model testing framework shown later in Table 2 and described here. In the first round of model competition, at‐site and between‐site models were competed separately. More specifically, the at‐site models were competed against an at‐site global model containing all at‐site landscape variables and a null model containing geographic distance alone. Separately, we conducted the same model competition among the between‐site models, a between‐site global model containing all between‐site variables, and a null model containing geographic distance alone. We ranked and selected models using Akaike information criterion scores, with ΔAIC <4 used as a threshold for model selection (Akaike, 1973; Burnham & Anderson, 2002). Next, models with ΔAIC <4 in the first round proceeded to a second round of model competition, again competing against a global model containing all landscape variables and a null model containing geographic distance. We used restricted maximum likelihood (REML) to calculate parameter estimates (Zuur et al., 2009) and confidence intervals (measured by Cohen's D) under the best‐fit model (Lakens, 2013). If the confidence interval excluded zero, that parameter was considered an important driver of the model (Zuur et al., 2009). As the variables are unscaled and log transformed, the magnitudes of the effect sizes are not directly comparable. Rather, the effect sizes indicate directionality of the components in the final model.

**TABLE 2:** Gravity model competition for at‐site, between‐site, and combined models based on the ecological processes described in Table 1. Results were estimated by maximum likelihood and ranked by ΔAIC values for at‐site and between‐site model comparisons. All models include geographic distance. At‐ and between‐site models with ΔAIC <4 were competed in the combined models. Starred (*) models were tested in the combined model competition.

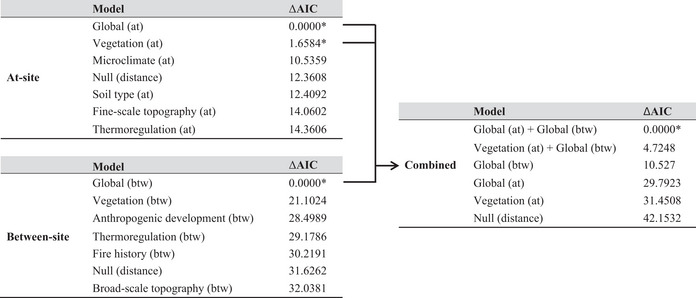

## 3 RESULTS

### 3.1 Data summary

BestRAD libraries for the 144 individual lizards in the study generated 969,708,736 and 976,705,586 raw reads for each lane of sequencing. After filtering, 743,210,602 and 750,437,524 reads (average per individual: 7,741,777 and 7,817,057) were retained. The lanes had average retained reads of 88.1% and 88.7%, indicating good alignment. The average final sequencing coverage (read depth per locus per individual) was 15x. Before applying allele filtering, we removed 10 individuals with high missing data (>95%). The final filtered population genetic data set contained 9448 SNPs for 134 individuals (Table S1).

### 3.2 Population genetic structure

PC1 explained 34.9% in our PCA, with samples from El Segundo Dunes clustering apart from the remaining individuals (Figure S2A). Franklin Canyon and Griffith Park individuals formed two distinct clusters, with San Rafael Hills individuals clustering more closely to those from Griffith Park. Verdugo Mountains individuals formed a wide cluster predominantly driving PC2. Samples were strongly clustered by sampling region; however, there was significant overlap among the Santa Monica Mountains, Simi Hills, San Gabriel Mountains, and Santa Susana Mountains. Removing individuals from El Segundo Dunes revealed a large amount of variation driven by the Verdugo Mountains, Franklin Canyon, and Griffith Park, and little separation among the remaining sampling regions (Figure S2B).

Population‐ and individual‐based metrics revealed high levels of genetic differentiation and strong structure across the study area. Pairwise population differentiation (*F*_ST_) ranged from 0.015 to 0.433 (average = 0.183) with all being significantly greater than zero (Table 3). Individuals from El Segundo Dunes were the most highly differentiated from all others, whereas the least differentiated were within the Santa Monica Mountains, Simi Hills, and Santa Susana Mountains. Based on the AMOVA, a significant amount of the variation in genotypes was explained by the geographic regions (17.7%) defined by coarse topography and habitat fragmentation, and there was significant variation within regions (Table 4).

**TABLE 3:** Pairwise differentiation (*F*_ST_) between the geographic regions delineated by topography and fragmentation of the study area. All comparisons are significant *(p* < .005).

	ESD	PIR	SGM	SIM	SMM	GRI	FRA	SSM	VER
ESD	—	—	—	—	—	—	—	—	—
PIR	0.433	—	—	—	—	—	—	—	—
SGM	0.369	0.025	—	—	—	—	—	—	—
SIM	0.431	0.048	0.059	—	—	—	—	—	—
SMM	0.418	0.038	0.05	0.015	—	—	—	—	—
GRI	0.224	0.195	0.145	0.203	0.199	—	—	—	—
FRA	0.354	0.213	0.168	0.211	0.193	0.192	—	—	—
SSM	0.419	0.041	0.049	0.027	0.017	0.189	0.201	—	—
VER	0.197	0.201	0.159	0.209	0.216	0.099	0.186	0.198	—

**TABLE 4:** Distribution of genetic variation within and among populations based on AMOVA, when “population” is the geographic region delineated by topography and fragmentation. Geographic regions were the following: Piru Creek, Santa Susana Mountains, Santa Monica Mountains, Simi Hills, Franklin Canyon, Griffith Park, Verdugo Mountains (including the San Rafael Hills), San Gabriel Mountains, and El Segundo Dunes.

Source of variation	Nested in	% var	*F*‐stat	*F*‐value	Std. dev.	c.i.2.5%	c.i.97.5%	*p*‐value	*F*′‐value
Within individual	–	57.8	*F*_IT_	0.422	0.002	0.419	0.426	‐‐	–
Among individual	Population	24.5	*F*_IS_	0.298	0.002	0.294	0.301	<.001	–
Among population	–	17.7	*F*_ST_	0.177	0.002	0.174	0.181	<.001	0.226

**FIGURE 2:**
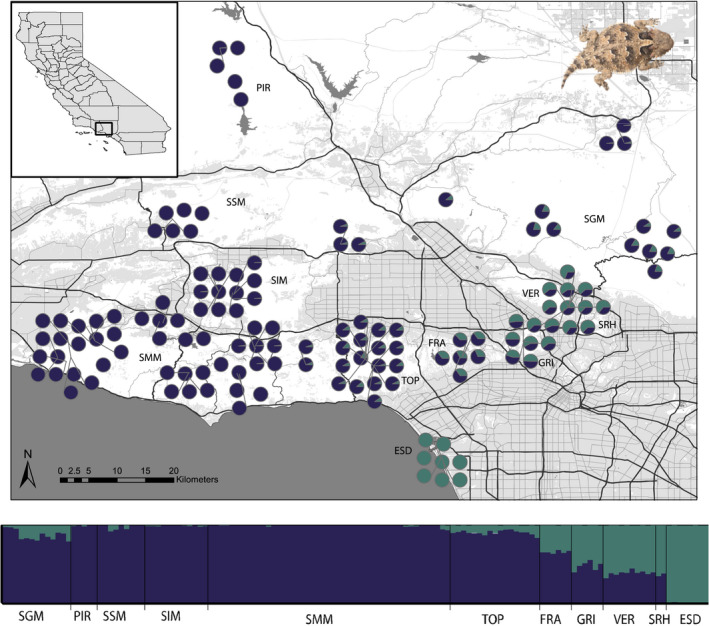
Population genetic structure (*K* = 2) of *Phrynosoma blainvillii* in the context of spatial relationships among individuals and urban development of the study area. Land data includes major roadways; undeveloped parks/natural open space (white); and commercial/industrial, residential, agricultural, and developed parks/other open areas (gray). Data sources: Southern California Association of Governments (2016) and Geographic Data Technology, Inc.

**FIGURE 3:**
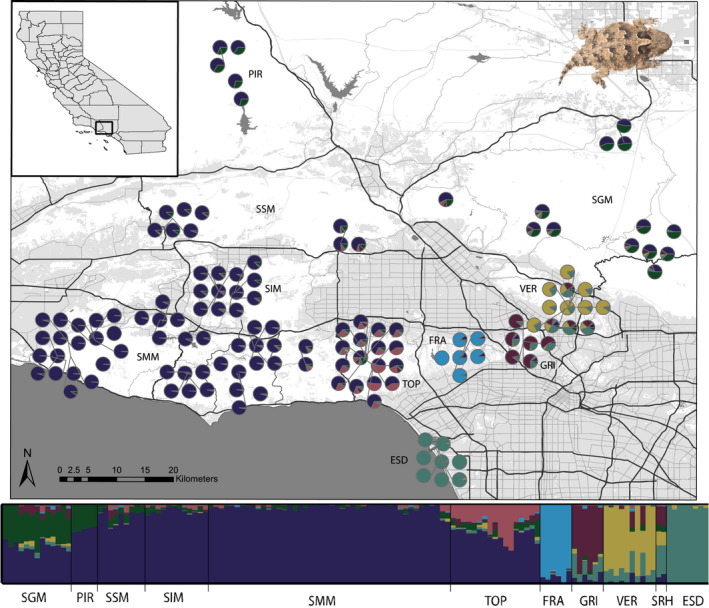
Population genetic structure (*K* = 7) of *Phrynosoma blainvillii* in the context of spatial relationships among individuals and urban development of the study area and in traditional structure bar plot. Land data includes major roadways; undeveloped parks/natural open space (white); and commercial/industrial, residential, agricultural, and developed parks/other open areas (gray). Data sources: Southern California Association of Governments (2016) and Geographic Data Technology, Inc.

Using cluster assignments for the full data set (134 individuals), we found population structuring at multiple scales. *K* and ln(Pr(X|*K*) support two and seven clusters, respectively, but we detected informative patterns at *K* = 2–7 (Figure S3). We report the assignments for *K* = 2 because the patterns differentiate between the northern and southern lineages, and *K* = 7 because it represents the finest level of population structure across the study area. When two ancestral populations were assumed, population structure occurred latitudinally, such that Piru Creek, Santa Susana Mountains, Simi Hills, and Santa Monica Mountains comprise one cluster, El Segundo Dunes formed a second cluster, and Verdugo Mountains, Griffith Park, and Franklin Canyon contained substantial admixture between the two (Figure 2). At *K* = 7, Verdugo Mountains, Franklin Canyon, and Griffith Park comprised individual clusters (Figure 3). We performed a subset of assignment tests on the two main clusters to examine substructure.

### 3.3 Gravity models

Both the 50‐ and 10‐km pruned networks achieved the same results, but the 10‐km pruned network improved model fit without changing model rankings (Naujokaitis‐Lewis et al., 2013). In addition, most edges of the 10‐km pruned network represent connections between individuals within the same geographic region, which is more biologically relevant. The best model of functional connectivity was the global–global model. This model contained all at‐site variables, all between‐site variables, plus geographic distance with site as a random effect. A subset of the model parameters had effect sizes with 95% confidence intervals that did not overlap zero (Table 5), and thus are considered important drivers in the model. These included % shrub, compound topographic index, heat load index, annual dryness index, and % sand for the at‐site model, and surface relief ratio (27 × 27 window), impervious surfaces, time since last fire, heat load index, and compound topographic index for the between‐site model. The directionality of Cohen's D was reversed for variables with negative values after log‐transformation and for between‐site variables due to the nature of the gravity model equation (see Table 5). Finally, geographic distance between sites had a negative relationship with gene flow, supporting genetic isolation by distance in this system.

**TABLE 5:** Parameter estimates based on Cohen's D for variables included in the final combined gravity model, global (at) + global (btw). Effect sizes with confidence intervals that span zero are considered insignificant. Effect size direction and corresponding interpretation account for sign changes due to log transformation.

	Variables	*t* value	Df	Cohen's D	*p*‐value	Lower CI	Upper CI	Effect size direction	Interpretation
At‐site	Geographic distance (Euclidean)	−3.638	1724	−0.1752	.0003	−0.2422	−0.1083	−	Geographic distance impedes gene flow
	Median surface relief ratio (3 × 3)	−1.3421	126	−0.2391	.1820	−0.4892	0.0109		No significant effect
	Percentage shrub land cover	−2.4917	126	−0.444	.0140	−0.6961	−0.1918	+	Higher proportion of native shrub promotes productivity
	Median compound topographic index	−2.2216	126	−0.3958	.0281	−0.6474	−0.1443	−	Moisture impedes productivity
	Median heat load index	0.3744	126	0.0667	.7087	−0.1825	0.3159		No significant effect
	Median annual drying index	1.7797	126	0.3171	.0775	0.0664	0.5678	+	Drier climate promotes productivity
	Median % sand in topsoil (0–5 cm)	2.45	126	0.4365	.0157	0.1844	0.6886	+	Higher sand content promotes productivity
	Median surface relief ratio (27 × 27)	−2.3702	1724	−0.1142	.0179	−0.181	−0.0473	−	Complex broad‐scale topography impedes gene flow
Between‐site	Median impervious surfaces	−2.8105	1724	−0.1354	.0050	−0.2023	−0.0685	−	Impervious surfaces impede gene flow
	Median time since last fire	2.7826	1724	0.134	.0055	0.0672	0.2009	−	Recent fire facilitates gene flow
	Median heat load index	2.9189	1724	0.1406	.0036	0.0737	0.2075	+	Greater incident radiation facilitates gene flow
	Median compound topographic index	−4.2578	1724	−0.2051	.0000	−0.2721	−0.1381	+	Moisture facilitates gene flow
	Percentage shrub land cover	−0.8763	1724	−0.0422	.3810	−0.109	0.0246		No significant effect

## 4 DISCUSSION

We examined the effects of environmental factors and anthropogenic disturbance on functional connectivity among populations of *P. blainvillii* in the highly developed Los Angeles region. We show that patterns of functional connectivity emerge from complex interactions between natural and anthropogenic environmental variation and microhabitat preferences. Specifically, we found that anthropogenic disturbances reduce connectivity among populations that are otherwise structured by biogeographical features and species ecology. Environmental factors limiting functional connectivity may become more imminent threats to population persistence under continued urban development and climate change. Uncovering how these factors operate on different time scales to create contemporary patterns of population structure is critical to develop management plans for species in urban‐impacted regions.

### 4.1 Biogeographic features shaped evolutionary divergence on regional scale

Divergence and introgression between the Northern California and Southern California evolutionary lineages can be explained by the historical biogeography of the Greater Los Angeles Area. The Northern California *P. blainvillii* lineage wraps around the San Fernando Valley of northern Los Angeles in a ring‐like pattern before introgressing with the Southern California lineage on the eastern side of that ring. Lineage divergence may have emerged in response to historically unsuitable habitat and/or heavy urbanization in the San Fernando Valley, as has been suggested by the lack of occurrence records across much of the lowland Los Angeles Basin (Brattstrom, 2013). Prior to urban development, stream corridors (e.g., Big Tujunga Creek) and soils with high sand content in the southeastern end of the San Fernando Valley (e.g., in the vicinity of North Hollywood) may have provided enough suitable habitat in the lowlands to facilitate limited genetic connectivity, producing the pattern of admixture seen at low *K* values in the structure analysis (Figure 2).

Along the coast, the Southern California *P. blainvillii* lineage reaches its northern extent at the El Segundo Dunes (ESD). Prior to the 1900s, sand dune habitat was abundant along much of the Los Angeles coastline from the Palos Verdes Peninsula in the south to Ballona Creek, which is immediately north of the ESD. Up until the 1860s, Ballona Creek was frequently the outflow for the Los Angeles River watershed (Gumprecht, 1999), suggesting that this major river and associated coastal wetlands (Dark et al., 2011) was a likely biogeographic break. When not flowing via the Ballona channel, the Los Angeles River flowed south as it does today. Sandy habitats existed along portions of this drainage, and *P. blainvillii* museum specimens demonstrate this species could be found through the 1950s up to 20 km inland near the lower Los Angeles River (e.g., LACM 101356–101,361). There are no historical records upriver from these localities, suggesting a distributional gap and lack of recent gene flow, but increased connectivity prior to urbanization may have facilitated limited gene flow between these coastal populations and those along the upper margins of the Los Angeles Basin at Griffith Park, the Verdugo Mountains, and the San Rafael Hills. Further north, the contact zone between the Northern and Southern California lineages through the San Gabriel Mountains remains unclear. Increased sampling in the eastern and central San Gabriel Mountains is needed to understand contact zone dynamics in this area (Figure 2).

At higher *K* values, population clusters in the Santa Monica Mountains have little to no shared ancestry across the Santa Monica slate, a geological feature that sits where the I‐405 freeway is today (Figure 3; Alvarez & Bovard, 2005). This pattern of genetic divergence combined with the lack of occurrence records within the area of Santa Monica slate suggests that this feature may have formed a historical barrier to gene flow before the landscape was modified by the freeway. Corroborating this idea, we found that populations in the eastern end of the Santa Monica Mountains (Franklin Canyon and Griffith Park), as well as the Verdugo Mountains and San Rafael Hills, were genetically unique from populations to the west (e.g., Topanga), on the opposite side of this feature (Figure 3). Therefore, populations in these four localities represent important components of genetic diversity with a complex evolutionary history established long before urbanization of the region.

### 4.2 Strong microhabitat preferences affect functional connectivity on a local scale

The ecology of *P. blainvillii* has influenced local scale patterns of functional connectivity. *Phrynosoma blainvillii* is a small‐ranging ectotherm with strong microhabitat preferences, including a dry microclimate, native scrub vegetation, and friable sandy soil. Proxies for these elements (annual dryness index, percent shrub, and percent sandy soil) were significant at‐site variables in the final gravity model, which represent conditions that facilitate *P. blainvillii* reproductive productivity (Table 5). Furthermore, the significant between‐site variables in our final gravity model indicate that broad‐scale topography, impervious surfaces, and time since fire inhibit *P. blainvillii* movement, while moisture and heat (compound topographic index and heat load index, respectively) facilitate movement among sites (Table 5). Environmental conditions have pronounced effects in shaping population structure for species with strong habitat preferences and/or ecological limitations, especially in areas where the intervening landscape is impenetrable to movement (e.g., Engler et al., 2014; Manel et al., 2003; Spear et al., 2005; Wang, 2009; and others).

Despite the naturally patchy distribution of *P. blainvillii* populations, the lack of population structure over the large geographic area of the western portion of the Santa Monica Mountains and to the north indicates historical connectivity (Figure 3). This panmixia despite the presence of contemporary barriers (such as the US‐101 freeway) can be explained by genetic inertia, probably due to large population sizes remaining in the mostly undeveloped parkland of the Santa Monica Mountains and in the northern Transverse Ranges. Identifying and conserving productive *P. blainvillii* local‐scale habitat areas within the larger landscape may be an important strategy for maintaining large and stable population sizes (see Conservation Management, below).

Contrary to the weak genetic structure in the western portion of the Santa Monica Mountains, a stronger signal of genetic structure among Topanga Canyon and surrounding localities to the east and west may signify the early stages of genetic divergence due to barriers on the landscape (Figure 3). Although the Santa Monica Mountains contain a large amount of contiguous habitat, there are heavily trafficked roads, several high gradient streams, and other barriers that partition the landscape. Horned lizard occurrence records based on National Park Service sampling and photo vouchers contributed to iNaturalist indicate a 6 km gap between Topanga Canyon and populations to the west in the Santa Monica Mountains, but more intensive sampling is needed to understand the causes of genetic divergence among horned lizards in this site and nearby populations.

### 4.3 Anthropogenic disturbance affects functional connectivity on rapid time scales

Anthropogenic disturbance regimes have complex effects on functional connectivity of *P. blainvillii*. Strong population structure among urban‐isolated habitat fragments and reduced connectivity across impervious surfaces suggests that *P. blainvillii* is susceptible to urban fragmentation effects (Figure 3; Table 5; Miles et al., 2019). Urban fragmentation presents several challenges for *P. blainvillii* beyond impeded connectivity, including reduced habitat quality along fragment edges, the spread of invasive Argentine ants, and increased predation risk by cats (Brattstrom, 2013). Additionally, *P. blainvillii* may be vulnerable to road mortality because it relies on crypsis, moves in short bursts, and tends to remain still when threatened (Thomson et al., 2016). The horned lizard localities that are most isolated by urbanization occur at the eastern end of the study area, encompassing the populations with complex evolutionary histories (see above). Of these, Franklin Canyon, Griffith Park, and the San Rafael Hills contain much smaller habitat areas and probably support very small population sizes of *P. blainvillii*. Therefore, the elevated levels of genetic differentiation among these populations are partially attributable to the demographic effects of habitat fragmentation (Table 2; DiLeo & Wagner, 2016; Fahrig, 2003; May et al., 2019). Despite the challenges of urban fragmentation on isolated populations, small fragments may still serve as important reserves for remnant populations (Delaney et al., 2021).

Changing disturbance regimes due to human‐activity are imminent threats to *P. blainvillii* population persistence. *Phrynosoma blainvillii* prefers open habitats, and landscapes that have been opened due to fire may facilitate movement (Rochester et al., 2010). Time since fire is negatively correlated to gene flow between sites (Table 5), indicating higher levels of gene flow through more recently burned landscapes. There is a complex relationship between horned lizards and fire. Fire may facilitate horned lizard movement by creating open ground, and harvester ants respond positively to fire due to the increased availability of larger seeds from invasive grasses (Matsuda et al., 2011; Mitrovich et al., 2010). However, short fire return intervals have detrimental effects due to direct mortality, loss of habitat, and vegetation community conversion (Rochester et al., 2010; Schrey et al., 2016; Smith et al., 2014). Decreasing fire intervals throughout Southern California are detrimental to native chaparral vegetation communities and have led to conversion to non‐native grasses when the interval is too short to permit regrowth of native shrubs (Keeley, 2000; Zedler et al., 1983). These nonnative grasses may limit movement in the stout‐bodied *P. blainvillii* and reduce the amount of suitable habitat. Therefore, though *P. blainvillii* may benefit from fires as a facilitator for gene flow over short time scales, the species suffers negative effects when fires are too frequent to permit the recovery of native vegetation. Under climate change scenarios, fire return intervals will continue to decrease, while this region will also experience increasing temperatures, longer droughts, and unseasonal rain events. Combined, these factors could degrade the long‐term persistence of this ecological specialist, especially in small fragments already isolated by urbanization.

### 4.4 Conservation management

Understanding the interplay of natural and anthropogenic landscape features in shaping patterns of functional connectivity may be critical to develop management strategies for this declining species. For example, strategies such as translocations or genetic rescue operate differently depending on the causes of divergence, with the long‐term strategy being to preserve historically rooted genetic divergence (to reduce risk of swamping local adaptation) while ameliorating loss of genetic diversity due to drift (Allendorf et al., 2013; Frankham et al., 2010). This may be an important issue in remnant populations occupying small and urban‐isolated geographic areas, including Griffith Park, Franklin Canyon, San Rafael Hills, and Verdugo Mountains. Treating them as independent management units is prudent given their restricted habitat areas and genetic exclusivity, and the reduced habitat quality along the urban edge. Future work should aim to determine whether genetic rescue is necessary for these urban‐isolated populations.

*Phrynosoma blainvillii* in the Greater Los Angeles region would benefit from immediate management actions promoting native vegetation, reducing encroachment by nonnative grasses, and increasing population monitoring. Though individuals in the Santa Monica Mountains and Santa Susana Mountains exhibit weak population structure, genetic divergence among these regions is likely to increase over time given the barriers in place. Anthropogenic effects on gene flow may not show for many generations depending on standing genetic diversity and effective population size (Johnson & Munshi‐South, 2017; Miles et al., 2019).

Potential long‐term management actions for horned lizards and other small‐ranging, ecological specialists include restoration of connectivity across major barriers in the region, as is currently underway through the construction of a wildlife crossing between the Santa Monica Mountains and the Simi Hills. Examining other diverse taxa, particularly those also incapable of using the urban matrix, is an important next step in developing management strategies in other biodiversity hotspots and urbanized regions (e.g., Tassone et al., 2020).

## 5 CONCLUSIONS

This research applies landscape genetics to enhance our understanding of the effects of environmental factors on contemporary functional connectivity of *P. blainvillii* in the Greater Los Angeles Area. Landscape genetics models that include measures of preferred habitat (at‐site variables), such as gravity models, may be particularly important for estimating functional connectivity for species with ecological specialization and/or patchy distributions, where the production of migrants is a critical component of gene flow (e.g., Murphy, Dezzani, et al., 2010; Zero et al., 2017). The use of these types of network models may have practical applications to species recovery, such as the identification of important habitat areas, and modelling climate change or other anticipated disturbances to facilitate proactive management. As the availability of genetic and environmental data increases, these tools can be applied in diverse taxa and environments to help guide conservation strategies and species recovery.

## AUTHOR CONTRIBUTIONS

Sarah M. Wenner, Kathleen S. Delaney, Gregory B. Pauly, Jonathan Q. Richmond, Robert N. Fisher, and Jeanne M. Robertson conceived and designed the study. The National Park Service and the U.S. Geological Survey provided samples, and Sarah M. Wenner and Gregory B. Pauly conducted additional field sampling in 2018 and 2019. Sarah M. Wenner performed the molecular laboratory work. Jeanne M. Robertson provided laboratory and computational resources for generating genetic data. Sarah M. Wenner and Melanie A. Murphy conducted data analysis. Sarah M. Wenner drafted the manuscript and all authors critically revised and approved the final version.

## CONFLICT OF INTEREST

The authors have no conflicts of interest to declare.

## Supporting information


Table S1
Figure S1
Figure S2
Figure S3

## Data Availability

Raw data, input files, and scripts have been made available in Figshare at https://doi.org/10.6084/m9.figshare.19658487 (Wenner et al., 2022).
