# Supplementary material for: Natural and anthropogenic landscape factors shape functional connectivity of an ecological specialist in urban Southern California
Source: Mol Ecol. 2022 Sep 13;31(20):5214–30. doi: 10.1111/mec.16656 (PMC9826396; doi:10.1111/mec.16656)

Supplemental Tables and Figures

Table S1. Region, locality, and sampling dates for *Phrynosoma blainvillii* included in final molecular analysis (*n = 134*). GPS coordinates available only upon request due to sensitivity of the species.

Individual ID	Geographic Area	Locality	Sampling Date
PHBL_129	El Segundo Dunes	Los Angeles Int'l Airport	2/29/16
PHBL_130	El Segundo Dunes	Los Angeles Int'l Airport	2/29/16
PHBL_362	El Segundo Dunes	Los Angeles Int'l Airport	4/3/17
PHBL_363	El Segundo Dunes	Los Angeles Int'l Airport	5/13/17
PHBL_399	El Segundo Dunes	Los Angeles Int'l Airport	4/13/17
PHBL_400	El Segundo Dunes	Los Angeles Int'l Airport	4/3/17
PHBL_401	El Segundo Dunes	Los Angeles Int'l Airport	4/13/17
PHBL_402	El Segundo Dunes	Los Angeles Int'l Airport	4/13/17
PHCO_473	San Gabriel Mountains	Alder Creek	5/1/11
PHBL_134	San Gabriel Mountains	Aliso Canyon	3/18/16
PHBL_136	San Gabriel Mountains	Aliso Canyon	3/18/16
PHBL_406	San Gabriel Mountains	Aliso Canyon	6/25/18
PHCO_467	San Gabriel Mountains	Gold Creek Saddle	4/22/18
PHCO_465	San Gabriel Mountains	Gold Creek Saddle	4/22/18
PHCO_466	San Gabriel Mountains	Gold Creek Saddle	4/22/18
PHCO_461	San Gabriel Mountains	May Canyon Saddle	6/9/18
PHCO_460	San Gabriel Mountains	Mt. Disappointment	4/14/18
PHCO_157	San Gabriel Mountains	Strawberry Peak	8/4/18
PHCO_113	San Gabriel Mountains	Strawberry Peak	8/4/18
PHCO_115	San Gabriel Mountains	Strawberry Peak	8/19/18
PHCO_469	San Gabriel Mountains	Upper Big Tujunga Canyon	4/11/15
PHCO_463	San Rafael Hills	San Rafael Hills	6/12/18
PHCO_468	San Rafael Hills	San Rafael Hills	6/19/18
PHCO_055	Santa Monica Mountains	Castro Crest	5/29/18
PHCO_065	Santa Monica Mountains	Castro Crest	5/29/18
PHCO_437	Santa Monica Mountains	Castro Crest	4/22/16
PHCO_105	Santa Monica Mountains	Franklin Canyon	4/26/19
PHCO_132	Santa Monica Mountains	Franklin Canyon	4/26/19
PHCO_136	Santa Monica Mountains	Franklin Canyon	4/26/19
PHCO_141	Santa Monica Mountains	Franklin Canyon	4/26/19
PHCO_153	Santa Monica Mountains	Franklin Canyon	4/17/19
PHCO_156	Santa Monica Mountains	Franklin Canyon	4/12/19
PHCO_056	Santa Monica Mountains	Griffith Park	4/9/18

PHCO_058	Santa Monica Mountains	Griffith Park	4/9/18
PHCO_066	Santa Monica Mountains	Griffith Park	4/9/18
PHCO_116	Santa Monica Mountains	Griffith Park	4/24/19
PHCO_143	Santa Monica Mountains	Griffith Park	4/24/19
PHCO_154	Santa Monica Mountains	Griffith Park	6/21/18
PHCO_067	Santa Monica Mountains	Liberty Canyon	4/10/18
PHCO_075	Santa Monica Mountains	Liberty Canyon	4/10/18
PHCO_078	Santa Monica Mountains	Liberty Canyon	4/10/18
PHCO_448	Santa Monica Mountains	Liberty Canyon	8/18/17
PHCO_449	Santa Monica Mountains	Liberty Canyon	8/18/17
PHCO_450	Santa Monica Mountains	Liberty Canyon	8/18/17
PHCO_451	Santa Monica Mountains	Liberty Canyon	8/18/17
PHCO_431	Santa Monica Mountains	Los Robles/Conejo Open Space	4/10/15
PHCO_452	Santa Monica Mountains	Los Robles/Conejo Open Space	8/20/17
PHCO_453	Santa Monica Mountains	Los Robles/Conejo Open Space	8/21/17
PHCO_454	Santa Monica Mountains	Los Robles/Conejo Open Space	8/22/17
PHCO_455	Santa Monica Mountains	Los Robles/Conejo Open Space	8/22/17
PHCO_422	Santa Monica Mountains	Malibu Creek SP	8/11/09
PHCO_009	Santa Monica Mountains	Point Mugu/La Jolla Valley	7/26/18
PHCO_401	Santa Monica Mountains	Point Mugu/La Jolla Valley	11/14/02
PHCO_404	Santa Monica Mountains	Point Mugu/La Jolla Valley	5/20/03
PHCO_405	Santa Monica Mountains	Point Mugu/La Jolla Valley	7/16/03
PHCO_406	Santa Monica Mountains	Point Mugu/La Jolla Valley	7/17/03
PHCO_407	Santa Monica Mountains	Point Mugu/La Jolla Valley	4/20/04
PHCO_408	Santa Monica Mountains	Point Mugu/La Jolla Valley	4/22/04
PHCO_409	Santa Monica Mountains	Point Mugu/La Jolla Valley	4/22/04
PHCO_410	Santa Monica Mountains	Point Mugu/La Jolla Valley	5/18/04
PHCO_419	Santa Monica Mountains	Point Mugu/La Jolla Valley	6/29/06
PHCO_420	Santa Monica Mountains	Point Mugu/La Jolla Valley	3/12/08
PHCO_421	Santa Monica Mountains	Point Mugu/La Jolla Valley	4/11/08
PHCO_426	Santa Monica Mountains	Point Mugu/La Jolla Valley	7/30/14
PHCO_438	Santa Monica Mountains	Point Mugu/La Jolla Valley	5/3/16
PHCO_440	Santa Monica Mountains	Point Mugu/La Jolla Valley	3/24/17
PHCO_444	Santa Monica Mountains	Point Mugu/La Jolla Valley	5/19/17
PHCO_445	Santa Monica Mountains	Point Mugu/La Jolla Valley	5/19/17
PHCO_447	Santa Monica Mountains	Point Mugu/La Jolla Valley	7/12/17
PHCO_060	Santa Monica Mountains	Puerco Canyon	5/21/18
PHCO_443	Santa Monica Mountains	Serrano Valley	5/5/17
PHCO_464	Santa Monica Mountains	Stunt Ranch	5/18/18
PHCO_475	Santa Monica Mountains	Stunt Ranch	5/18/18

PHCO_001	Santa Monica Mountains	Topanga Canyon	3/29/18
PHCO_002	Santa Monica Mountains	Topanga Canyon	3/29/18
PHCO_003	Santa Monica Mountains	Topanga Canyon	4/24/18
PHCO_004	Santa Monica Mountains	Topanga Canyon	4/24/18
PHCO_007	Santa Monica Mountains	Topanga Canyon	6/25/18
PHCO_008	Santa Monica Mountains	Topanga Canyon	6/29/18
PHCO_423	Santa Monica Mountains	Topanga Canyon	4/10/13
PHCO_424	Santa Monica Mountains	Topanga Canyon	6/30/14
PHCO_425	Santa Monica Mountains	Topanga Canyon	7/1/14
PHCO_430	Santa Monica Mountains	Topanga Canyon	3/27/15
PHCO_433	Santa Monica Mountains	Topanga Canyon	5/20/15
PHCO_436	Santa Monica Mountains	Topanga Canyon	4/6/16
PHCO_446	Santa Monica Mountains	Topanga Canyon	6/14/17
PHCO_456	Santa Monica Mountains	Topanga Canyon	10/29/17
PHCO_457	Santa Monica Mountains	Topanga Canyon	10/20/17
PHCO_462	Santa Monica Mountains	Topanga Canyon	6/11/18
PHCO_476	Santa Monica Mountains	Topanga Canyon	4/7/19
PHCO_063	Santa Monica Mountains	Trancas/Encinal Canyon	4/11/18
PHCO_068	Santa Monica Mountains	Trancas/Encinal Canyon	5/28/18
PHCO_076	Santa Monica Mountains	Trancas/Encinal Canyon	5/28/18
PHCO_080	Santa Monica Mountains	Trancas/Encinal Canyon	5/17/18
PHCO_103	Santa Monica Mountains	Triunfo Canyon	7/2/18
PHCO_146	Santa Monica Mountains	Triunfo Canyon	6/29/18
PHCO_069	Santa Monica Mountains	Zuma Ridge Mtwy	3/31/18
PHCO_071	Santa Monica Mountains	Zuma Ridge Mtwy	4/4/18
PHCO_073	Santa Susana Mountains	Happy Camp Canyon	4/21/18
PHCO_077	Santa Susana Mountains	Happy Camp Canyon	4/22/18
PHCO_131	Santa Susana Mountains	Happy Camp Canyon	7/31/18
PHCO_470	Santa Susana Mountains	Happy Camp Canyon	6/29/14
PHCO_471	Santa Susana Mountains	Happy Camp Canyon	6/25/14
PHCO_472	Santa Susana Mountains	Happy Camp Canyon	6/25/14
PHCO_104	Santa Susana Mountains	Michael D. Antonovich RP	9/13/18
PHCO_118	Santa Susana Mountains	Michael D. Antonovich RP	8/15/18
PHCO_139	Santa Susana Mountains	Michael D. Antonovich RP	8/17/18
PHCO_005	Simi Hills	China Flats	6/15/18
PHCO_006	Simi Hills	China Flats	6/15/18
PHCO_147	Simi Hills	China Flats	6/1/18
PHCO_428	Simi Hills	Lang Ranch/Albertson Motorway	3/13/15
PHCO_429	Simi Hills	Lang Ranch/Albertson Motorway	3/13/15
PHCO_432	Simi Hills	Lang Ranch/Albertson Motorway	5/5/15

PHCO_439	Simi Hills	Lang Ranch/Albertson Motorway	5/19/16
PHCO_441	Simi Hills	Lang Ranch/Albertson Motorway	4/6/17
PHCO_442	Simi Hills	Lang Ranch/Albertson Motorway	4/6/17
PHCO_458	Simi Hills	Lang Ranch/Albertson Motorway	4/5/18
PHCO_459	Simi Hills	Lang Ranch/Albertson Motorway	4/19/18
PHCO_474	Simi Hills	Lang Ranch/Albertson Motorway	3/29/19
PHBL_407	Topa Topa Mountains	Piru Creek	6/27/18
PHBL_408	Topa Topa Mountains	Piru Creek	6/27/18
PHBL_409	Topa Topa Mountains	Piru Creek	6/28/18
PHBL_410	Topa Topa Mountains	Piru Creek	6/29/18
PHBL_411	Topa Topa Mountains	Piru Creek	4/10/18
PHCO_106	Verdugo Mountains	Verdugo Mountains	6/15/18
PHCO_111	Verdugo Mountains	Verdugo Mountains	6/15/18
PHCO_120	Verdugo Mountains	Verdugo Mountains	6/15/18
PHCO_121	Verdugo Mountains	Verdugo Mountains	6/15/18
PHCO_123	Verdugo Mountains	Verdugo Mountains	6/15/18
PHCO_135	Verdugo Mountains	Verdugo Mountains	6/15/18
PHCO_142	Verdugo Mountains	Verdugo Mountains	6/15/18
PHCO_144	Verdugo Mountains	Verdugo Mountains	6/15/18
PHCO_148	Verdugo Mountains	Verdugo Mountains	6/15/18
PHCO_149	Verdugo Mountains	Verdugo Mountains	6/15/18

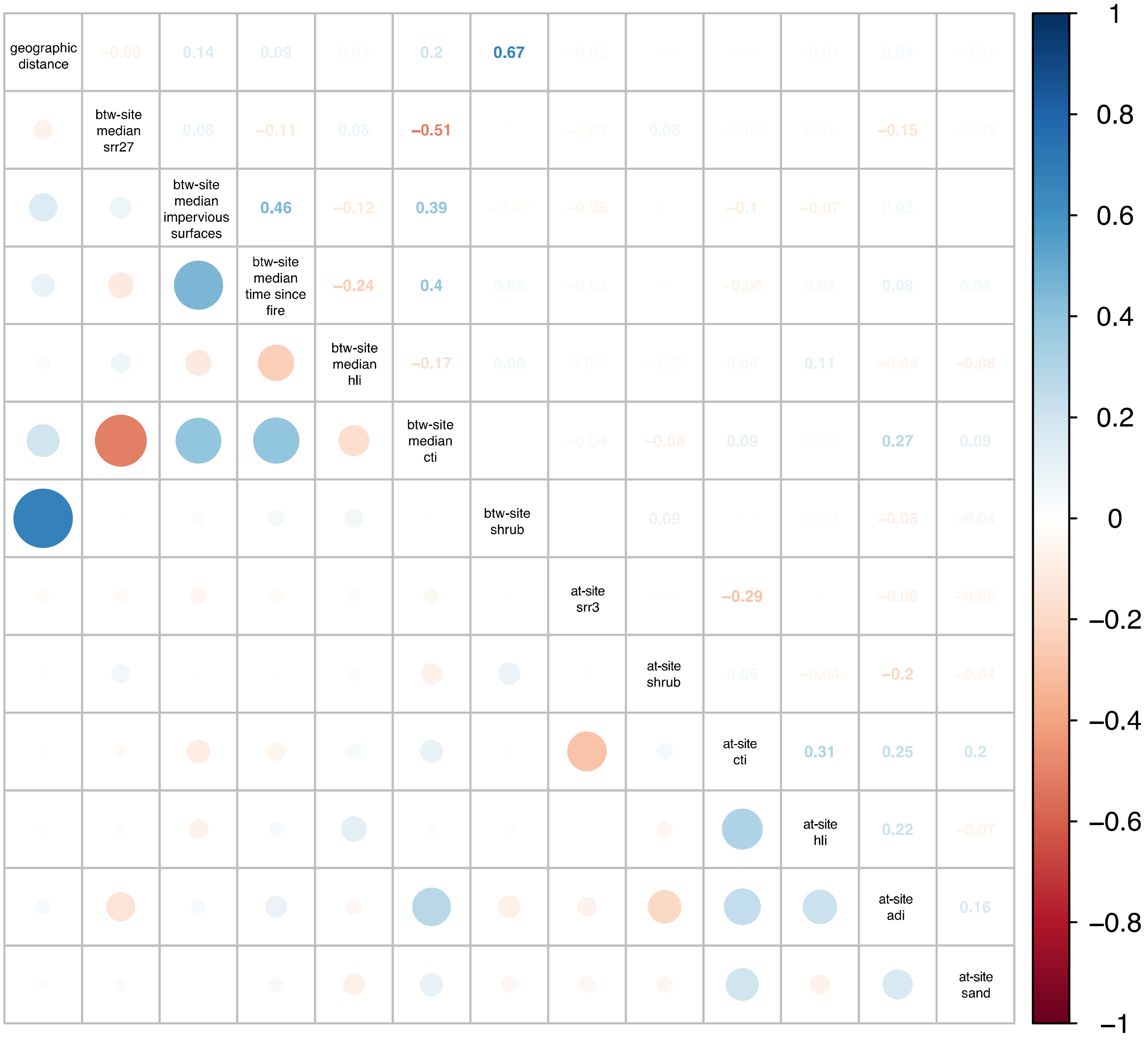
geographic distance
btw-site median srr27
btw-site median impervious surfaces
btw-site median time since fire
btw-site median hli
btw-site median cti
btw-site shrub
at-site srr3
at-site shrub
at-site cti
at-site hli
at-site adi
at-site sand
0.14
0.2
0.67
−0.51
−0.15
0.46
−0.12
0.39
−0.1
−0.24
0.4
−0.17
0.11
0.27
0.09
−0.08
−0.29
−0.2
0.31
0.25
0.2
0.22
0.16
1
0.8
0.6
0.4
0.2
0
−0.2
−0.4
−0.6
−0.8
−1

Figure S2. Principal component analysis plots of 9448 SNPs from (A) all samples and (B) all samples with El Segundo Dunes samples removed. Individuals are colored by geographic region and ellipses represent 95% confidence intervals for each grouping. San Rafael Hills individuals were combined with Verdugo Mountains individuals due to close geographic proximity and low sample size.

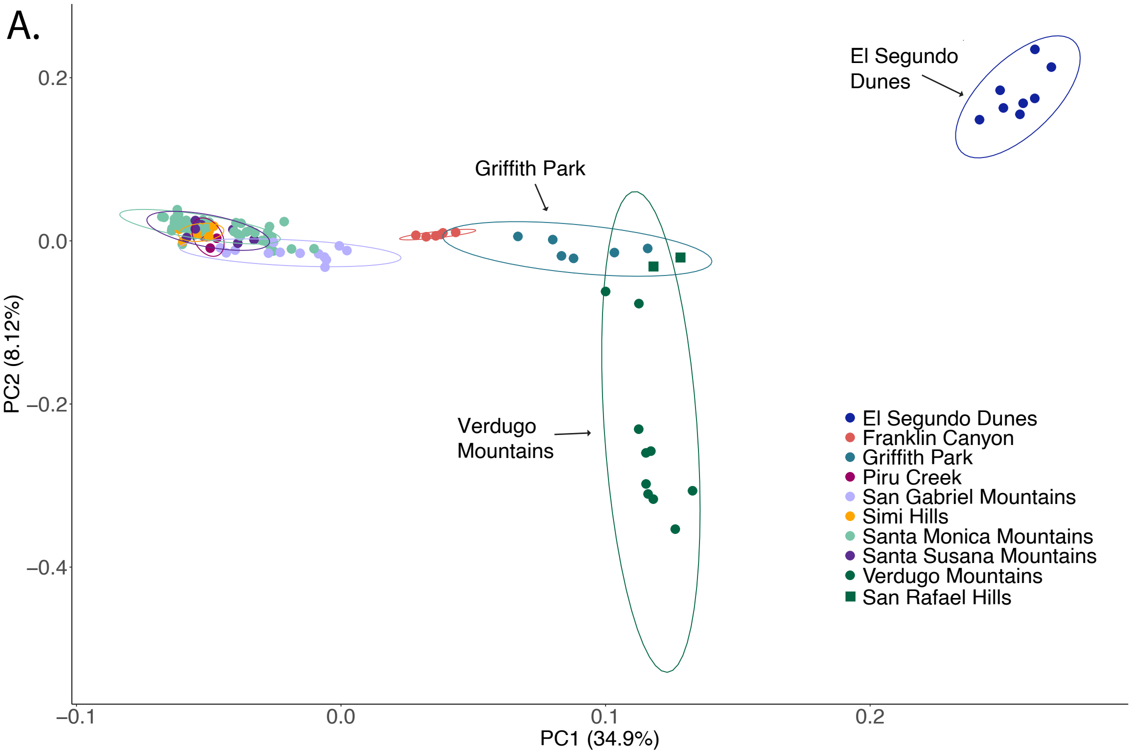

B.

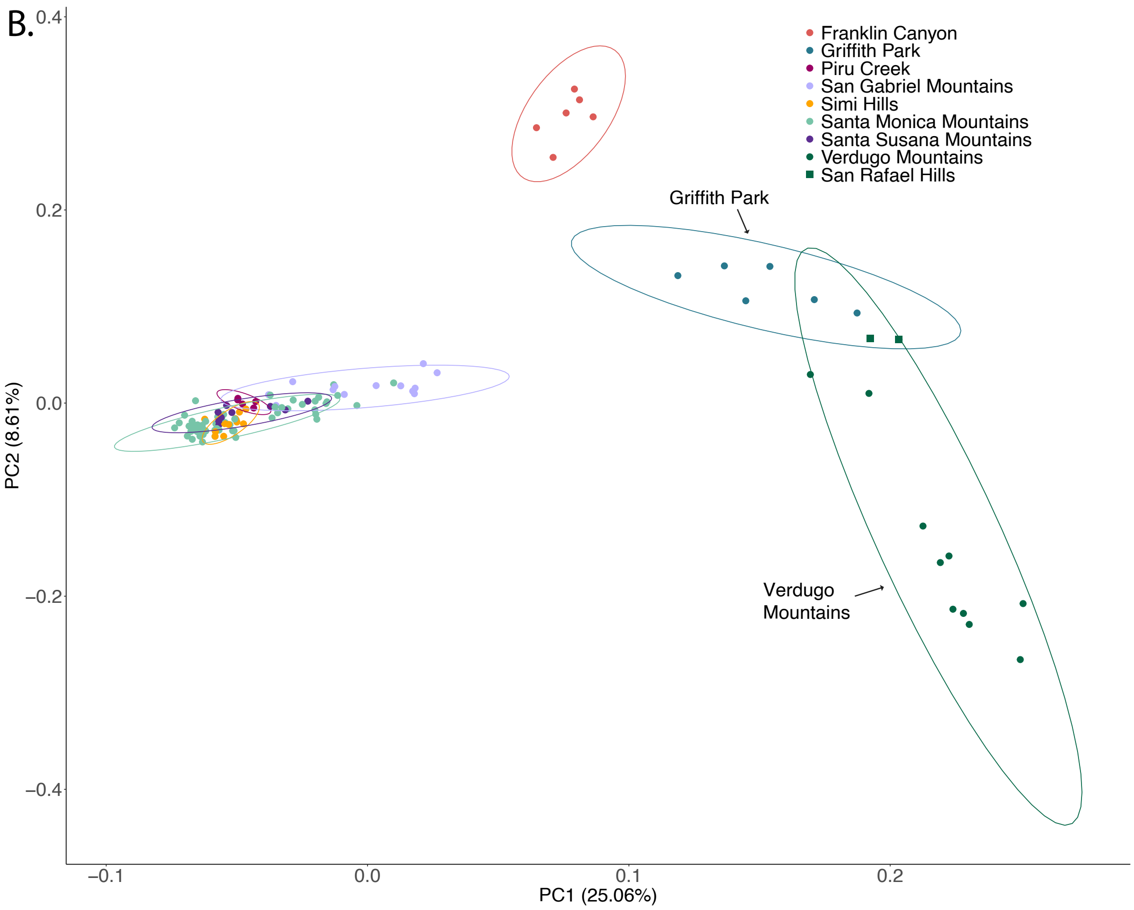
Franklin Canyon
Griffith Park
Piru Creek
San Gabriel Mountains
Simi Hills
Santa Monica Mountains
Santa Susana Mountains
Verdugo Mountains
San Rafael Hills
Griffith Park
Verdugo Mountains
PC2 (8.61%)
PC1 (25.06%)
0.4
0.2
0.0
−0.2
−0.4
−0.1
0.0
0.1
0.2

Figure S3. Population genetic structure of *Phrynosoma blainvillii* sampled in Los Angeles and Ventura counties. Best K as estimated by (A) ΔK and (B) ln(Pr(X|K)). Bar plot visualization of population genetic structure when (C) two and (D) seven genetic demes are assumed.

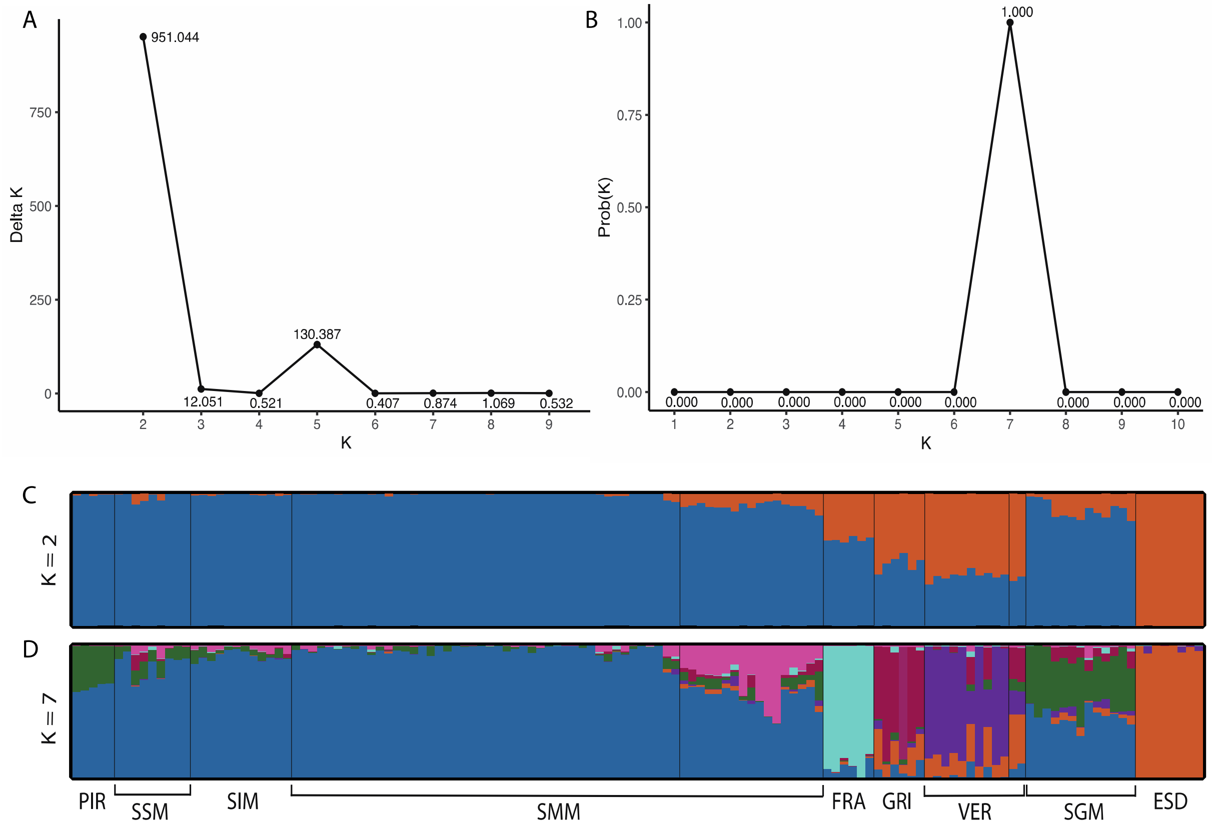

Supplement: Supplementary file 1 — Table S1 Figure S1 Figure S2 Figure S3 [file MEC-31-5214-s001.pdf]
